# Early rise of West Nile fever in Israel, June 2024

**DOI:** 10.2807/1560-7917.ES.2024.29.30.2400457

**Published:** 2024-07-25

**Authors:** Zohar Mor, Husein Omari, Victoria Indenbaum, Oscar D Kirstein, Oren Shatach Catabi, Shay Reicher, Yaniv Lustig, Maya Davidovich-Cohen, Ehud Kaliner, Rivka Sheffer, Shirly Elbaz, Or Kriger, Sharon Alroy-Preis

**Affiliations:** 1 Division of Epidemiology, Ministry of Health, Jerusalem, Israel; 2 School of Health Science, Ashkelon Academic College, Ashkelon, Israel; 3 Central Virology Laboratories, Ministry of Health, Tel Hashomer, Israel; 4 Public Health Laboratories, Ministry of Health, Jerusalem, Israel; 5 Ministry of Environmental Protection, Jerusalem, Israel; 6 School of Public Health, Tel Aviv University, Tel Aviv, Israel; 7 Central Department of Health, Ramla, Israel; 8 Tel Aviv Department of Health, Tel Aviv, Israel; 9 Public Health Directorate, Ministry of Health, Jerusalem, Israel

**Keywords:** Avian, Migratory birds, Mosquitoes, Neuroinvasive disease, One Health, West Nile virus, West Nile fever, Israel

## Abstract

This report describes an unusual surge of West Nile fever in Israel in June 2024, during which 125 cases were diagnosed, compared with 4 cases on average during June in previous years (2014–23). Of the cases, 64 (62.1%) had neuroinvasive disease and 12 (9.6%) died; the 2024 case fatality rate was not significantly elevated vs the average rate in 2014–23. The early rise could be related to a temperature increase in spring and early summer of 2024.

Israel is an endemic country for West Nile virus (WNV) since 1951 [1], and West Nile fever (WNF) cases are usually reported from June to October, with a peak in August. Here, we report a remarkable increase in WNF cases in early summer 2024 in Israel. We describe the epidemiological, clinical and genetic findings and compare to data reported in previous years (2014–23) and outline the public health measures to contain the outbreak using a One Health approach.

## Case definition

Demographic, epidemiological and clinical data for this study were collected from hospital records and epidemiological investigations. In Israel, WNF cases are defined as having compatible symptoms, e.g. fever, malaise, headache, neurological symptoms, and presence of one of the following laboratory results from 1 January 2014 to 30 June 2024: (i) WNV RNA in blood, urine or cerebrospinal fluid (CSF) detected by PCR; (ii) anti-WNV IgM in the CSF; (iii) seroconversion in two different blood samples indicated by the presence of anti-WNV IgM or IgG; or (iv) fourfold increase in the convalescent sample by a micro-neutralisation assay.

## Case characteristics

**Figure 1:**
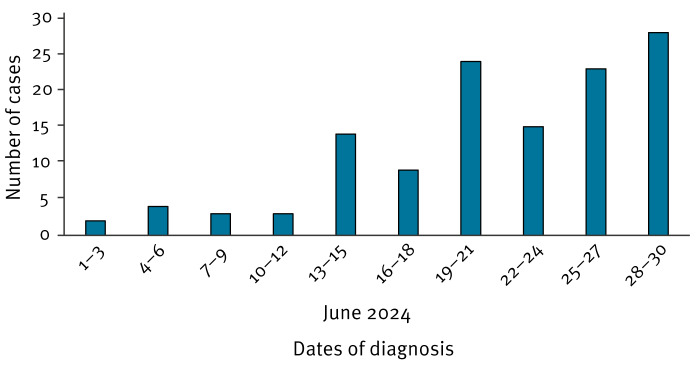
Epidemiological curve of cases with West Nile fever diagnosed in Israel, June 2024 (n = 125)

**Figure 2:**
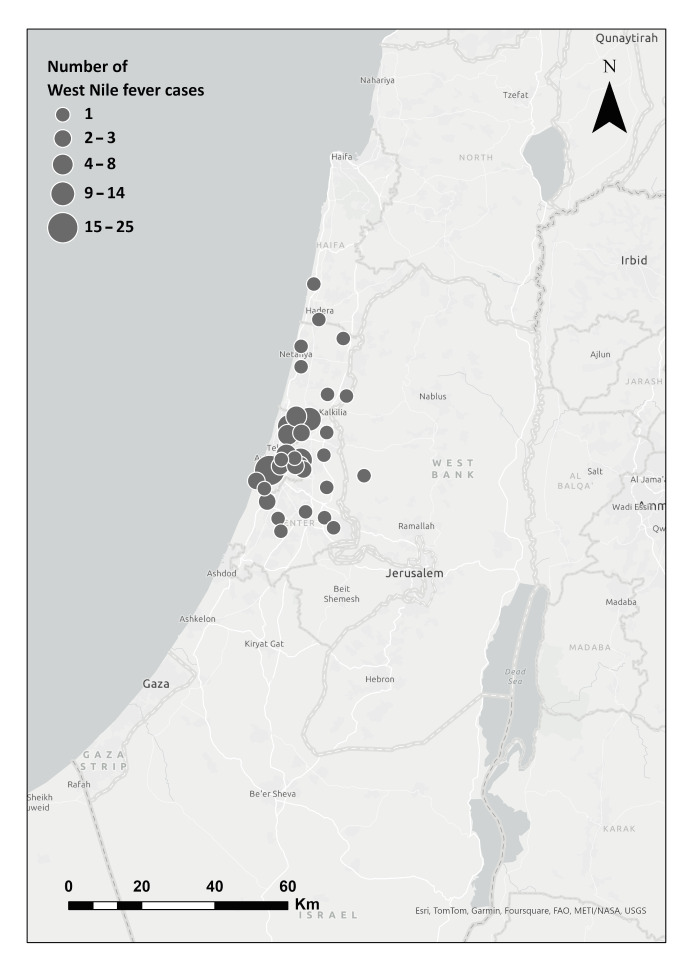
Geographic distribution of cases with West Nile fever diagnosed in Israel, June 2024 (n = 125)

In 2024, up to 30 June, 125 human cases of WNF were confirmed in Israel (Figure 1). Most were males (n = 73; 58.4% with n = 52; 41.6% females), and the median age was 71 years (interquartile range (IQR): 50–79); all cases lived in central Israel (Figure 2). Clinical presentation of 103 (82.4%) cases for which data were available included fever (n = 90; 87.4%), rash (n = 46; 44.7%), myalgia (n = 54; 52.4%) and neuroinvasive disease (n = 64; 62.1%). Eighty-three (66.2%) were hospitalised, of those 8 (9.7%) were ventilated mechanically and 12 of all 125 died (case fatality rate (CFR): 9.6%).

**Figure 3:**
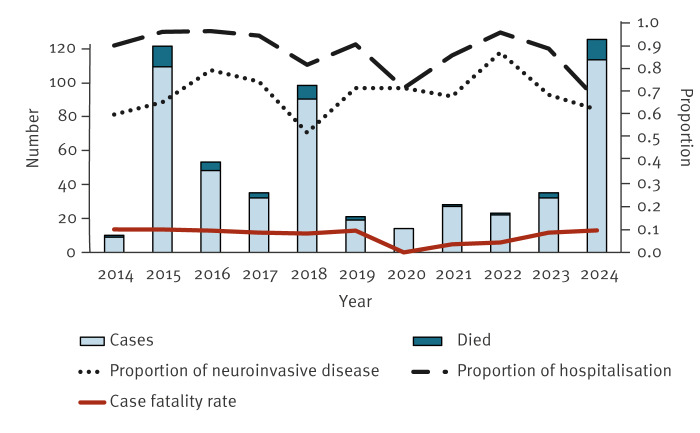
Number of cases with West Nile fever and proportion of hospitalisations, neuroinvasive disease and mortality, Israel, 2014–2024 (n = 561)

The 125 cases diagnosed in 2024 were compared with all 436 cases diagnosed between 2014 and 2023 (Figure 3). Those who were diagnosed in 2024 were more likely to have presentation of rash and showed lower hospitalisation rates than those diagnosed in 2014–23. No statistically significant differences were found in the male:female ratio, clinical presentation of fever, rate of neuroinvasive disease or CFR between the 2024 cases and those diagnosed between 2014 and 2023 (Table).

Annual data are presented for years 2014–23. Data for 2024 include all cases reported up to 30 June 2024.

**Table:** Comparison of demographic and clinical characteristics between cases with West Nile fever diagnosed in Israel in 2024 (n = 125) and in 2014–2023 (n = 436)

Characteristics	Cases diagnosed in 2024 (n = 125)		Cases diagnosed 2014–23 (n = 436)		p
	n	%	n	%	
Cases recorded Jan–Jun	125	100.0	61	13.9	< 0.001
Male:female ratio	1.4		1.5		0.2
Median age in years (IQR)	71 (50–79)		65 (47–75)		0.2
Fever^a^	90	87.4	383	87.8	0.9
Rash^a^	46	44.7	94	21.6	< 0.001
Neuroinvasive disease^a^	64	62.1	292	67.0	0.1
Hospitalised	83	66.4	395	90.6	< 0.001
Case fatality rate	12	9.6	32	7.3	0.4

IQR: interquartile range.

^a^ Clinical data were available for 103 of 125 confirmed cases.

Chi-square tests were used to compare categorical variables and the Student's t-test was used to compare continuous variables. P values greater than 5% were considered statistically significant.

Data for 2024 include all cases reported up to 30 June 2024.

## Entomological and genetic investigations of human and mosquito samples

Vector surveillance is routinely performed in Israel by capturing mosquitoes from traps which are situated in designated sites around the country. Of 279 traps in 2024, 10.4% (n = 29) had mosquitoes infected with WNV vs 0.65% (6/925) in the months of June each year between 2014 and 2023 (p < 0.01). The minimal infection rate, which is the number of positive pools of mosquitoes divided by the total number of vectors tested was 4.31 ((83/19,273)*1,000) in June 2024 compared with an average of 0.16 ((10/60,939)*1,000) in the months of June each year between 2014 and 2023 (p < 0.01).

Sanger sequencing of three randomly selected human samples and two mosquito pools from 2024 that were positive by quantitative RT-PCR for WNV demonstrated that all samples belonged to WNV lineage 1, clade 1a, cluster 2 (data not shown). The molecular results of samples from June 2024 were similar to the viruses detected in Israel in previous years [2]. 

## Public health interventions

As in 2024, WNV in Israel was also detected early in mosquitoes and birds in central Israel, routine monitoring efforts were intensified to enhance detection of geographic areas with WNF cases, mosquitoes and birds, as part of an integrated One Health strategy. All addresses of the human cases, along with the locations of the traps with infected mosquitoes and the sites where dead birds were found were immediately shared with the Ministry of Environmental Defence. The Ministry of Environmental Defence implemented environmental interventions, which included larviciding activities in areas where WNV-positive mosquitoes were present and in the peridomestic area/environment of infected individuals’ residences. In addition, the Ministry of Health alerted the public and provided recommendations for personal prevention activities, such as personal pest control measures, installing screens in homes and eliminating standing or stagnant water in the houses and yards.

## Discussion

West Nile virus is an arbovirus that was first identified in Uganda in 1937 and is transmitted in an enzootic cycle that flows mainly through urban dwelling of *Culex pipiens* and *perexiguus* mosquitoes and certain bird species [2]. Humans and mammals may be incidentally infected through mosquito bites, but they represent dead-end hosts. Meteorological conditions, such as the interplay between rainfall and high temperature influences mosquito breading and is associated with WNV transmission dynamics.

During the past 30 years, several outbreaks of WNV have been reported in humans and animals, with two peaks in 2018 and 2022 in Europe [3], and from 1999 in the United States [4]. WNV was first detected in Israel in 1951 [1] and has been notifiable in our country since 2001 [5]. The disease incidence in Israel has been characterised by sporadic outbreaks every few years [2,6,7], and the largest outbreak was recorded in 2000, with more than 440 cases and CFR of > 10% [8]. Genetic analysis attributed to most human, avian and mosquitoes sampling in Israel were mainly lineage 1, and to a lesser extent lineage 2 [9]. Around 20% of all WNF cases will present clinical symptoms and < 1% show neurological complications [10]. The CFR of neuroinvasive disease is around 10% [10], increasing with age and immunocompromised state. Although this report presents a significant surge of cases with WNV infection in Israel in June 2024 compared with previous years, no statistically significant differences were found in epidemiological characteristics and the proportion of fever, neuroinvasive disease and CFR.

Climate is one possible explanation for this early rise in WNF cases in June 2024 in Israel. The average temperature in central Israel during the month of June 2024 was 31.5 °C, which was 3 °C warmer than the average measurements performed between 1991 and 2020 [11]. Unusual precipitation of 6.8 mL was also recorded on 6 May (May monthly average 1991–2017: 0.9 mL), followed by several heatwaves in the first 2 weeks of June. Exceptionally warm spring temperatures along with rain showers may lead to earlier emergence and increased breeding activity, amplify the replication lifecycle, activity, biting and reproduction rates of *Culex* and are the driving forces behind WNF outbreaks [12-15]. Similarly, the viral establishment, replication and incubation period of mosquitoes are governed by temperature. Israel is located at a junction of three continents and serves a crossroad of migrating birds between Africa and Eurasia, especially in spring and autumn. The interaction between migrating birds, local avian species and mosquitoes augments the risk for WNV transmission.

Our study has several limitations. Firstly, the retrospective nature of the data from previous years may limit the comparison of some clinical features of WNF. Secondly, given a rise in public interest and physicians' awareness, mild cases were reported in 2024, which may have underestimated the proportion of neuroinvasive disease, hospitalisation rate and CFR relative compared to previous years. Finally, the precise clinical diagnosis of neuroinvasive disease (encephalitis, meningitis, etc.) was not available in some cases, and thus may not fully describe the severity of the neurological symptoms.

## Conclusion

Israel experienced a significant and unusual surge of WNF cases in early summer 2024, which could be related to a temperature increase in spring and early summer. As the clinical presentation and the CFR in 2024 were similar to those reported in 2014–23, we may conclude that the outbreak in 2024 is characterised by an early surge rather than a different pattern of virus or greater clinical severity. Implementation of public health and environmental measures that integrate surveillance of human cases, mosquito and avian species are important for effective prevention and control of WNF and other vector-borne diseases.
